# Unaccompanied refugee minors in Germany: attitudes of the general population towards a vulnerable group

**DOI:** 10.1007/s00787-017-0943-9

**Published:** 2017-01-10

**Authors:** Paul L. Plener, Rebecca C. Groschwitz, Elmar Brähler, Thorsten Sukale, Jörg M. Fegert

**Affiliations:** 1 Department of Child and Adolescent Psychiatry and Psychotherapy, University of Ulm, Steinhoevelstr. 5, 89075 Ulm, Germany; 2 Department of Medical Psychology and Medical Sociology, University Leipzig, Leipzig, Germany; 3 Department of Psychosomatic Medicine and Psychotherapy, University Medical Center, Johannes Gutenberg University Mainz, Mainz, Germany

**Keywords:** Unaccompanied refugee minors, URM, Refugee, Adolescents, Trauma, Flight

## Abstract

Germany saw an increase in numbers of refugees in 2015, with nearly a third being below the age of 18. Unaccompanied refugee minors (URMs) present an especially vulnerable group. In addition to pre-flight and flight stress, the acculturation process can work as potential stressor, and we wanted to explore attitudes towards URM. We conducted a study in a representative sample (*n* = 2524) of the German population (ages 14 years or older) between January and March 2016. Only 22.8% of participants thought that Germany could accompany more URM. While few participants argued in support of immediate deportation of URM in general (38.6%) or of URM from the Middle East (35.3%), a majority advocated for immediate deportations of URM from the Balkan region (62%) or from Africa (51.1%). Difference in the variance regarding attitudes towards deportation was explained mostly by right-wing political attitudes as well as by islamophobic attitudes and general rejection of asylum seekers. High rates of approval were found for guaranteeing the same chances to schooling or apprenticeship for URM as to German children and for bestowing URM a right to permanent residence if they were able to complete school or apprenticeship. Education and qualification are key to integration. Studies about needs and wishes of URM consistently report a high motivation to learn the language of their new host country and attend school. At this point, hopes of URM and expectations of society meet, which underlines the importance of participation in education as key factor in integration.

## Background

Among people seeking refuge, adolescents and young adults present a majority and it has been estimated that around 50% of refugees worldwide are below the age of 18 [1]. Germany saw a steep rise in the numbers of refugees in the year 2015, with the federal office for migration and refugees reporting that among them 31.1% were below the age of 18 and 26.5% below the age of 16 [2]. Among them were many unaccompanied refugee minors (URMs), defined as persons under 18 years of age seeking refuge in a foreign country without an adult being responsible for them [for definition, see 2, 3]. Although the large majority (86%) of refugee minors travel with their parents [4], recently, a large number of URM came to Europe, with their numbers in Germany increasing tenfold within 2 years [4]. In 2015, 14,439 applications for asylum were handed in by URM, of which 28.7% were handed in by URM younger than 16 years of age and 71.3% from the age group 16–18. With regard to the country of origin, the largest groups of URM came from Afghanistan (32.9%), Syria (27.6%), Eritrea, and Iraq (9.3% each) [2]. Not all URMs enter the registration in European countries and the EUROPOL has warned that many URMs seem to disappear from asylum or reception centers [5] and are used for labor exploitation. In addition, according to the International Organization for Migration (IOM) and UNICEF, there is a group of especially young, male asylum seekers, who avoid registration to escape protection measures and are at an especially high risk for exploitation [6]. With regard to legal aspects, it is noteworthy that URMs also include minors, who came to their new host countries through human trafficking [7].

### Traumatic experiences and consequences

Many of these refugee minors have experienced potentially traumatic events in their countries of origin (pre-flight experiences) and have been exposed to life-threatening or dangerous situations throughout their migration (flight experiences) [8]. In addition to these events, acculturation stress, difficulties with the integration, hostility, multiple moves, and separation from their families could increase the burden on these children in their new country of residence [4, 8, 9]. Furthermore, there are also reports of other risk factors for mental disorders in URM, such as domestic violence, not related to experiences of migration. In a recent study, Mueller-Barmough et al. [10] reported a history of domestic violence in 91.8% of a sample of 49 URM assessed in Germany.

Due to the lack of a family support system, which could potentially buffer stress, as well as accumulation of traumatic experiences, URMs have to be considered as especially vulnerable group [11, 12].

In determining the prevalence of mental health disorders among URM, studies using clinical interviews stated rates between 41 and 56%, with a high percentage of posttraumatic stress disorder (PTSD) (20–30%) [3]. The severity of PTSD is also influenced by feelings of guilt and shame, especially pronounced in URM [13]. Besides PTSD, there is also a high prevalence of affective or anxiety disorders [3, 14]. Comparing 920 URM to 1294 accompanied adolescent refugees and 1059 Dutch adolescents, a study from the Netherlands reported most internalizing symptoms in URM (while Dutch adolescents showed the highest rates of externalizing symptoms in this study) [15]. Supporting these findings, a recent study on 191 male URM in German youth welfare institutions reported rates of internalizing symptoms to be as high as 61% [16].

The most recent study from Germany based on data from a child and adolescent psychiatric outpatient clinic (*n* = 75) reported rates of mental disorders to be 75% [17], which seems high, compared to a rate of 20.2% of mental health problems, reported from children and adolescents in the German general population [18]. However, as URMs were sent to this outpatient clinic from a clearing site, this rate refers to a selected group of URM [17]. Of the URM with a psychiatric disorder, 98% had witnessed at least one traumatic event and 41% the killing of a family member [17].

A recent systematic review reported especially high rates (up to 97% in some studies) of potentially traumatizing events in URM, thus resulting in a high “trauma load” [3], with a recent study from German refugee minors showing that accompanied refugee minors reported a mean of three traumatic events, while URM reported a mean of seven events [13].

Still, little is known about the long-term course of mental disorders in the group of URM. In a study of 75 URM in Norway, a 1.9 year follow-up was conducted, showing stability of symptoms of depression, anxiety, and externalizing problems over time [19]. A study following 103 URM in Belgium for 18 months also reported rather stable level of psychiatric disorders. In addition to the number of traumatic experiences, the number of daily stressors after they reached their new country of residence also had a severe impact on levels of depression, PTSD, and anxiety [20].

Despite a high burden of potentially traumatizing life events, a great number of URM seem to be mentally stable and present themselves as “resilient”, not showing symptoms of a mental disorder, with rates between 44 and 58% in different studies [3].

### Attitudes towards refugees

In a survey conducted by the Allensbach Institute for Demoscopic Research in May 2015 [21], 1453 individuals in Germany (above the age of 16) were asked whether a further intake of refugees to Germany seemed possible, which was answered positively by 31% of the participants. Including country of origin, participant’s acceptance was higher for refugees from Syria or Iraq (31%), when asked, if Germany should accept as much refugees from these countries as possible. In comparison, less people were in favor of accepting as much refugees as possible from Africa (23%). Participants were further asked if they would support initiatives to build a refugee asylum in their place of residence, which was supported by 31%, a higher rate of approval than in a former survey (24%), conducted in 2014 [17]. In a follow-up study conducted in October 2015 (*n* = 1209), at a timepoint, when many refugees had entered Germany over the summer, rates of approval for hosting refugees in the region the participants lived in were as high as 54% [22]. Still, 32% of the participants responded positively to the question whether Germany would be capable to accept more refugees. However, many participants answered that they were very worried (54%) or somewhat worried (38%) about the development of the refugee situation in Germany [22].

While in 2015, in Germany, “welcome culture” was a political slogan accepted by large parts of the general population proud about this humanistic attitude, recently the debate shifted to issues, such as better border controls and potential islamistic terrorist threats. In general, accepted standards of youth welfare for URM have been questioned by high-ranking politicians. Most child welfare organizations and advocacy groups as well as the German scientific society of child and adolescent psychiatry psychosomatics and psychotherapy appealed against this proposal [23].

Given that the acculturation process in a new country can act as a potential stressor, and daily stressors have a severe impact on URM’s mental health [20], we wanted to explore general attitudes towards URM in the general population of Germany. In trying to find explanations for attitudes towards URM, we assessed demographic factors as well as political positions. Furthermore, we aimed at assessing attitudes towards key aspects of integration, such as schooling, apprenticeship, or housing. To the best our knowledge, this is the first study to present data on this subject.

## Method

This study was conducted to gather information about attitudes towards URM from a representative sample (*n* = 2524) of the German population (ages 14 years or older) between January and March 2016. Using a random route method, participants were visited at home and informed about the study by 228 research assistants. After providing informed consent, a demographic interview was conducted and paper and pencil questionnaires were handed to the participants. These questionnaires were collected later on. Out of 4830 households that were approached, 1541 were not available (after four approaches) or did not respond, 14 persons were of ill health or not able to fill out the questionnaires, and 731 refused to participate. Out of 2544 collected questionnaires, 20 were not analyzable, thus resulting in 2524 participants (52.3%).

Based on the aforementioned questions regarding attitudes towards refugees [21], we adapted the items to assess attitudes towards UMR, asking if participants thought that Germany will be able to host more URM (Is Germany capable of hosting more URM?). Participants were able to answer positively, negatively, or could state that they were undecided on that issue. To assess attitudes towards URM from different regions of origin we asked, whether URM (in general, from the Balkan peninsula, from Africa, and from the Middle East) should be sent back to their home countries. Answers could be provided on a four-point Likert scale (fully agree-fully opposed). Regarding schooling or apprenticeship, we asked whether URM should have the same rights to access schooling or apprenticeship as adolescents with German nationality, also providing a four-point Likert-scale answering scheme (fully agree–fully opposed). The same answer format was used when asking if URM should be allowed to stay in Germany if they have finished either school or apprenticeship in Germany. Our last question pertained to the housing situation of URM, assessing where participants thought that URM should live (with pre-defined answer categories being based on the actual situation in Germany: in refugee housing, in youth welfare homes, and in foster care or “elsewhere”).

To assess political attitudes and attitudes to asylum seekers, in general, we used a validated measure of political right-wing extremist attitudes (Fragebogen zum Rechtsextremismus-Leipziger Form: FR-LF). This 18 item scale consists of six subscales (approval of dictatorship, chauvinism, hostility to foreigners, anitsemitism, socialdarwinism, and belittlement of nationalsocialism) with three items/subscale. This scale has been used in bi-annual representative population surveys in Germany from the year 2002 to detect trends in right-wing extremism attitudes [24–26]. Furthermore, we used assessment of islamophobia and rejection of asylum seekers in general (with two items each) as proposed in Decker et al. [25]. Participants were asked to rate themselves on a political spectrum from left to right wings on a ten-point Likert scale [25, 26], so that we were both able to assess right-wing extremism (by the FR-LF scale) and political attitudes (using the ten-point Likert scale).

The survey was in concordance with the Declaration of Helsinki met ethical guidelines of the international code of Marketing and Social Research practice by the International Chamber of Commerce and the European Society for Opinion and Marketing Research and was approved by the Institutional Review Board of the Medical Faculty of the University of Leipzig. Statistical analysis was performed using IBM SPSS, Vers. 21.

## Results

Only 22.8% agreed that it is possible for Germany to take in more URM, with no significant gender difference (*χ* ^2^: 5.19; *p* = 0.075). There was a tendency to a more positive attitude in younger participants with lower rates of approval in older participants. Furthermore, participants, who did not have a German citizenship, more often agreed to the statement that Germany has the potential to accept more URM (39.8 vs. 22.1%; *χ* ^2^: 21.67, *p* < 0.001). In participants with a lower monthly income after taxes, more negative attitudes were present (*χ* ^2^: 15.78, *p* = 0.003). Having graduated from high school was associated with a more positive attitude (*χ* ^2^: 99.04, *p* < 0.001) (see Table 1 for details).

**Table 1:** Is Germany capable of hosting more URM? Analyses based on gender, age, and nationality (*n* = 2524) ^a^ missing: 70

Is Germany capable of hosting more URM?	Capable (%)	Not capable (%)	Undecided/no answer (%)
Gender			
Male (%)	259 (22.8)	542 (47.8)	333 (29.4)
Female (%)	310 (22.8)	596 (43.9)	453 (33.3)
Total (%)	569 (22.8)	1138 (45.6)	786 (31.5)
Age			
Up to 24 years	73 (26.1)	117 (41.8)	90 (32.1)
25–34 years	98 (27.2)	158 (43.9)	104 (28.9)
35–44 years	91 (24.5)	159 (42.9)	121 (32.6)
45–54 years	115 (23.9)	229 (47.5)	138 (28.6)
55–64 years	95 (20.8)	224 (49.1)	137 (30.0)
65–74 years	58 (17.7)	155 (47.4)	114 (34.9)
Ab 75 years	39 (18.0)	96 (44.2)	82 (37.8)
Nationality			
German	528 (22.1)	1110 (46.4)	752 (31.5)
Non-German	41 (39.8)	28 (27.2)	34 (33.0)
High school graduation			
No	365 (18.6)	966 (49.2)	631 (32.2)
Yes	204 (38.4)	172 (32.4)	155 (29.2)
Monthly income (after taxes)^a^			
No income	31 (23.8)	52 (40.0)	47 (36.2)
Income < €1.500	280 (20.3)	674 (48.9)	425 (30.8)
Income ≥ €1.500	239 (26.1)	386 (42.2)	289 (31.6)

Differentiating between regions of origin of URM and asking whether URM from different parts of the globe should be sent back, an interesting pattern emerged. Although few people fully agreed to sending back URM from the middle east, more than half of the participants (fully) agreed that URM from the Balkan peninsula or from Africa should be sent back to their home countries. There was a gender effect with females being more clearly opposed to sending back URM to their home country in general (*χ* ^2^2: 15.20, *p* = 0.002) to the Balkans (*χ* ^2^2: 27.76, *p* < 0.001), Africa (*χ* ^2^2: 26.23, *p* < 0,001), and the Middle East (*χ* ^2^2: 33,40, *p* < 0.001) (Table 2).

**Table 2:** Should URM be sent back to their home countries (in general and differentiated per region of origin) (*n* = 2499)

Should URM be sent back to their home countries?	Fully agree			Somewhat agree			Somewhat oppose			Fully oppose		
	M (%)	F (%)	Total (%)	M (%)	F (%)	Total (%)	M (%)	F (%)	Total (%)	M (%)	F (%)	Total (%)
All URM (%)	175 (15.4)	152 (11.2)	327 (13.1)	293 (25.7)	343 (25.2)	636 (25.5)	422 (37.1)	497 (36.5)	919 (36.8)	249 (21.9)	368 (27.1)	617 (24.7)
URM from the Balkans (%)	408 (35.7)	390 (28.8)	798 (31.9)	341 (29.8)	412 (30.4)	753 (30.1)	287 (25.1)	344 (25.4)	631 (25.3)	107 (9.4)	210 (15.5)	317 (12.7)
URM from Africa (%)	277 (24.4)	251 (18.6)	528 (21.2)	313 (27.6)	406 (30.0)	719 (28.9)	374 (33.0)	408 (30.2)	782 (31.5)	169 (14.9)	287 (21.2)	456 (18.4)
URM from Middle East (%)	191 (16.9)	157 (11.6)	348 (14.0)	221 (19.5)	308 (22.8)	529 (21.3)	436 (38.5)	443 (32.8)	879 (35.4)	284 (25.1)	443 (32.8)	727 (29.3)

**Fig. 1:**
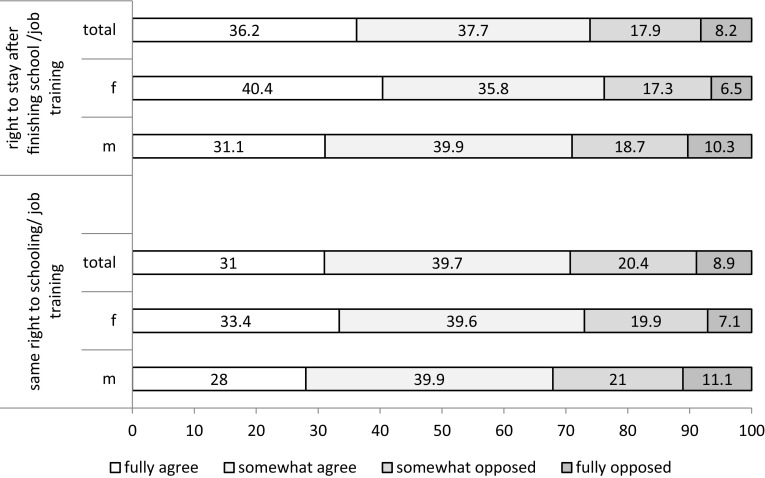
Agreement (in percent) regarding the right to stay in Germany after finishing school or job training and regarding granting the same school/job training opportunities to URM than children with German nationality (*n* = 2494)

Looking into integration, questions regarding schooling/job training and housing were asked. There was a large majority both for granting URM the same schooling or apprenticeship possibilities as adolescents from Germany and for allowing URM with a finished school exam and job training to stay in Germany (see Fig. 1). Female participants agreed even stronger to URM being granted the same schooling or job training possibilities (*χ* ^2^2: 17.0, *p* = 0.001) and the right to stay in Germany after finishing school or a job training (*χ* ^2^2: 26.19, *p* < 0.001).

Asked in which place URM should live, most participants were in favor of youth welfare homes (44.6%), followed by refugee housings (29.2%) and foster care (18.5%) (elsewhere: 7.7%). This results in more than half of the participants approve of offering the same options to URM (youth welfare institutions and foster care) than to all other children in need in Germany.

### Regression analyses

The variables gender, age, income, migration background, level of education, political attitudes (left- or right-orientation), right-wing extremism, islamophobia, and rejection of asylum seekers were entered into regression analyses.

In a binary stepwise regression analysis, the question whether participants thought Germany was capable of taking on more URM (yes/no) was examined. A model, including the variables right-wing extremism, islamophobia, and rejection of asylum seekers, explained 44.3% of the variance. Islamophobia and rejection of asylum seekers predicted disagreement most strongly (OR 2.1; 95% CI 1.8–2.6 and OR 2.1; 95% CI 1.7–2.7, respectively), followed by right-wing extremism (OR 1.1; 95% CI 1.0–1.1). All other variables were excluded from the model due to insignificance. For further details, see Table 3.

**Table 3:** Regression analyses on attitudes towards URM

	Wald	OR (95% CI)
Is Germany capable of hosting more URM (y/n)?		
Right-wing extremism	67.96***	1.05 (1.04–1.06)
Islamophobia	65.48***	2.14 (1.78–2.58)
Rejection of asylum seekers	41.37***	2.14 (1.70–2.70)

	*T*	*β*
Should URM be sent back to their home country immediately (1 = totally agree, 4 = totally disagree)?		
Gender	1.52	0.02
Age	−1.95	−0.03
Income	0.18	0.00
Migration background	0.18	0.00
Level of education	1.53	0.03
Political attitudes	−2.58**	−0.04
Right-wing extremism	−10.68***	−0.22
Islamophobia	−17.99***	−0.39
Rejection of asylum seekers	−8.32***	−0.16
Should URM from the Balkan peninsula be sent back immediately (1 = totally agree, 4 = totally disagree)?		
Gender	3.23***	0.06
Age	−4.32***	−0.08
Income	−2.07*	−0.04
Migration background	2.71**	0.05
Level of education	0.30	0.01
Political attitudes	−2.36*	−0.05
Right-wing extremism	−5.54***	−0.13
Islamophobia	−10.66***	−0.26
Rejection of asylum seekers	−10.77***	−0.23
Should URM from Africa be sent back immediately (1 = totally agree, 4 = totally disagree)?		
Gender	2.29*	0.04
Age	−3.59***	−0.06
Income	0.12	0.00
Migration background	0.52	0.01
Level of education	−1.47	−0.03
Political attitudes	−1.57	−0.03
Right-wing extremism	−9.45***	−0.20
Islamophobia	−15.39***	−0.34
Rejection of asylum seekers	−11.39***	−0.22
Should URM from the Middle East be sent back immediately (1 = totally agree, 4 = totally disagree)?		
Gender	1.71	0.03
Age	−1.95	−0.03
Income	−0.17	−0.00
Migration background	−1.35	−0.02
Level of education	−0.33	−0.01
Political attitudes	−1.59	−0.03
Right-wing extremism	−9.85***	−0.20
Islamophobia	−19.77***	−0.43
Rejection of asylum seekers	−6.41***	−0.12
Should URM have the same right to receive education as German youth (1 = totally agree, 4 = totally disagree)?		
Gender	−2.08*	−0.04
Age	−1.70	−0.03
Income	1.29	0.03
Migration background	−0.52	0.03
Level of education	0.16	0.00
Political attitudes	2.61**	0.05
Right-wing extremism	3.52***	0.09
Islamophobia	7.97***	0.21
Rejection of asylum seekers	7.15***	0.17
Should URM be allowed to stay in Germany after completing education in Germany (1 = totally agree, 4 = totally disagree)?		
Gender	−3.32**	−0.06
Age	−0.39	−0.01
Income	−0.78	−0.02
Migration background	−0.07	−0.00
Level of education	−0.56	−0.01
Political attitudes	2.45*	0.05
Right-wing extremism	2.28*	0.06
Islamophobia	7.98***	0.21
Rejection of asylum seekers	7.06***	0.16

*OR* odds ratio [EXP (B)], *CI* confidence interval, *β* beta (standardized coefficient)

* *p* < 0.05, ** *p* < 0.01, *** *p *< 0.001

In logistic stepwise regression analyses, the questions whether participants thought URMs should be sent back immediately, also in differentiation by origin countries of the URM, and questions regarding schooling or apprenticeship possibilities were analyzed.

A model, including the variables political attitudes, right-wing extremism, islamophobia, and rejection of asylum seekers, explained 44.0% of the variance to the question whether URMs should be sent back immediately (Likert scale from 1 = totally agree to 4 = totally disagree). Islamophobia (*β* = −0.4, *T* = −18.0, *p* < .001), right-wing extremism (*β* = −0.2, *T* = −10.7, *p* < 0.001), rejection of asylum seekers (*β* = −0.2, *T* = −18.3, *p* < 0.001), and political attitudes (*β* = −0.04, *T* = −2.6, *p* = 0.01) significantly predicted attitudes towards this question.

Outcomes of logistic stepwise regression analyses differed by country of origin of URMs. While islamophobia, rejection of asylum seekers, right-wing extremism, political attitudes, age, gender, migration background, and income significantly predicted the question whether URMs from the Balkan peninsula should be sent back immediately (model explaining 30.4% of the variance), the same question regarding URMs from Africa was significantly predicted by islamophobia, rejection of asylum seekers, right-wing extremism, age and gender (model explaining 41.0% of the variance), and the same question regarding URMs from the Middle East was only significantly predicted by islamophobia, rejection of asylum seekers, and right-wing extremism (model explaining 42.0% of the variance). For further details, see Table 3.

The variables islamophobia, rejection of asylum seekers, right-wing extremism, political attitudes, and gender significantly predicted both questions whether URMs should have the same right to receive education as German youth (model explaining 17.0% of the variance) and whether URMs should have the right to stay in Germany if they completed their education in Germany (model explaining 15.7% of the variance). For further details, see Table 3.

## Discussion

Only a fifth (22.8%) of participants thought that Germany could accommodate more URM, whereas 45.6% responded negatively. This is quite a low rate, especially in comparison with the studies from Petersen [21] and Koecher [22], who reported rates of 31 or 32%, respectively, to a comparable question asking whether Germany would be capable to welcome refugees in general. There are two possible explanations for this rather low rate. One could be the time of the survey. While the studies of Petersen [21] and Koecher [22] were conducted in August and October 2015, our study was conducted between January and March 2016. At this time, there might have been a shift in the public opinion about refugees, following the events on new year’s eve in Cologne, where a mass sexual harassment was linked by media to a high number of immigrants [27, 28], thus creating a more hostile attitude. Furthermore, it might well be that the attitude against URM is more negative in comparison with the attitude against refugees in general, also including families and—when looking into gender issue—more girls. As accompanied refuge minors show lower rates of male adolescents in comparison with URM (50 vs. 86%), our finding could also be interpreted as a more opposing attitude to a predominantly male refugee group, also in light of the aforementioned events of sexual harassment and the associated media coverage and the perceived menace of terrorist attacks in European countries.

We found a differentiation for countries of origin when asking for immediate deportation as often claimed by right-wing populists. While fewer participants argued in support of immediate deportation of URM in general (38.6%) or of URM from the Middle East (35.3%), a majority advocated for immediate deportations of URM from the Balkan region (62%) or from Africa (51.1%). Of note, regression analysis revealed that attitudes to deportation were influenced by islamophobic tendencies and a general negative attitude towards asylum seekers. Gender and age only predicted attitudes towards deportation of youth from the Balkans or Africa, with generally more negative attitudes towards URM in male and in older participants. This might be due to the fact that there are no current war zones in the Balkans and these countries of origin have been declared as “safe” by the German administration. However, this is not true for a large number of countries or regions in Africa. It might be that our questions were to crude to differentiate between peaceful African nations and warzones, and it would be advisable to assess this distinction in future studies. It seems hardly understandable why islamophobic tendencies influenced tendencies towards deportation of URM from the Balkan peninsula, as only Bosnia and Hercegovina, and Albania and Kosovo have a muslim majority population. However, it might be that this result is due to over-generalization.

Looking into explanations for attitudes towards deportation, we assessed both demographic variables as well as political attitudes. In doing so, we saw that political attitudes more leaning to the right spectrum, right-wing extremism, a general rejection of asylum seekers as well as islamophobic attitudes influenced the attitudes towards URM.

The finding that right-wing political attitudes led to a lower acceptance of URM are in line with a study of 216 US students, also showing that a more conservative political attitude led to lower levels of acceptance regarding the intake of more refugees than a more liberal attitude [29].

We saw that a higher level of education was associated with more positive attitudes towards URM, and it seems warranted to state that education holds the key to a more welcoming and accepting attitude towards URM. This is a finding often encountered in research on attitudes towards immigration per se [30].

With regard to integration of URM already in Germany, high rates of approval where found for guaranteeing the same chances to schooling or apprenticeship for URM as to German children. This is in line with Article 22 of the Convention of children’s rights [31]. In addition, nearly, three quarters of participants were in favor of bestowing URM a right to permanent residence if they were able to complete school or apprenticeship. This should inform politics, showing that besides a reluctance against accepting more URM, austerity and depriving URM of rights to education are not acceptable for a large majority.

Although it has been shown that psychiatric disorders in URM seem to remain stable over time [20], some factors have the potential to increase mental health. In a study on 895 URM, who had stayed in Norway on average 3.5 years, support from the family had a positive influence on depressive symptomatology. Furthermore, also social support from friends had effects on levels of depression that were mediated by host cultural competence and discrimination [32]. As integrating URM in school or apprenticeship could lead to an increase in host cultural competence and decrease discrimination, these factors could have an influence on the mental health burden of URM.

It seems that education and qualification hold the key to integration based on a large consensus in German society. Of note, studies about needs and wishes of URM consistently report a high motivation to learn the language of their new host country and attend school [3]. At this point, hopes of URM and expectations of society meet, which underlines the importance of participation in education as key factor in integration.

### Limitations

Although our study presents a representative sample of the German population, some limitations apply: (1) although answers were provided in questionnaire that was handed back in a sealed envelope, it might well be that we encountered a social desirability bias, shifting answers towards a more positive attitude towards URM. However, this process still seems preferable to—for example—telephone surveys, who are even more prone to this bias. (2) Due to the sampling method, some individuals could not be reached, especially those in institutions or homes and those not literate in German. These factors may have influenced representativity. (3) The evaluation was not based on a validated assessment scale. However, we decided to apply questions used in further large studies on the attitudes towards refugees in Germany [17, 18] to be able to compare our results to these studies, which—given the timing of our study after the large number of refugees that entered Germany in 2015—seemed to be of interest.

Overall, to the best of our knowledge, this is the first study on attitudes towards drawing from a nationwide representative sample. Despite the low rates of approval for accepting more URM, the need for offering opportunities to URM and integrating them into society seems to be based on a broad consensus in the general population that contradicts political attempts to lower standards of youth welfare and to deny young refugees over the age of 18 any educational or apprenticeship perspective.
